# Human trafficking and labor exploitation: Toward identifying, implementing, and evaluating effective responses

**DOI:** 10.1371/journal.pmed.1002740

**Published:** 2019-01-29

**Authors:** Ligia Kiss, Cathy Zimmerman

**Affiliations:** Department of Global Health and Development, London School of Hygiene & Tropical Medicine, London, United Kingdom

## Abstract

In an Editorial, Ligia Kiss and Cathy Zimmerman discuss the need for research on the prevention of human trafficking and mitigation of its effects.

Global estimates suggest that about 25 million people are subjected to “modern slavery” in the form of forced labor or human trafficking [1]. These men, women, and children are often migrant workers who are exploited in diverse sectors, such as agriculture, mining, fishing, factory work, domestic work, and forced sex work [1,2]. Although the eradication of modern slavery is among the 2030 Sustainable Development Goals [3], development of effective responses for trafficking prevention and assistance for victims remains elusive in this nascent field of health research. We believe that intensified efforts against trafficking require a greater understanding of modifiable factors and the causal pathways that lead to trafficking in different contexts and for individual populations.

Human trafficking frequently involves multiple forms of abuse, including deception, coercion, extortion, threats, and, for many, physical or sexual violence. A growing body of research shows that survivors of extreme exploitation often suffer severe and enduring health consequences [4–7]. Trafficking is associated with physical injuries including fractures, lacerations and lost limbs [5,8], chronic pain and headaches, significant weight loss [7,9], and symptoms of infectious and chronic diseases [8]. Sexual and reproductive health problems are common among women who are sexually exploited and abused while trafficked [4,9]. For trafficking survivors, persistent health problems include mental health consequences, especially symptoms of post-traumatic stress disorder, depression, anxiety, and suicidality [4,5,9,10].

Despite the significant health burden of human trafficking, only recently have health professionals begun to engage in responses to trafficking [2,11]. Findings from the study of violence against women suggest that healthcare providers are often a first nonfamily point of contact for victims of abuse. Recognizing that a healthcare setting can be a unique opportunity for well-trained providers to identify, assist, and refer trafficking survivors to necessary services [8,12,13], some governmental and intergovernmental agencies have begun to develop guidance. For example, the United Kingdom Department of Health has invested in research to support medical responses [9], whereas the United States Department of Health and Human Services recently launched the SOAR (i.e., Stop, Observe, Ask, Respond) training course [8,14], and international training tools are available to support healthcare providers to care for trafficked persons [13].

From a policy perspective, there has been disappointingly little engagement with modern slavery as a health concern by government health departments, such as health ministries, or by international agencies, including WHO. Evidence for prevention strategies is still scarce—particularly intervention-focused research and evaluations [13–16]. Given the scale of the problem and concomitant harms, human trafficking and modern slavery should be treated as a global health concern. Prevention and intervention approaches should, therefore, draw on and learn from approaches and methods used in the evaluation of other population health risks such as violence, smoking, and obesity.

In the first generation of research on human trafficking and modern slavery, efforts focused primarily on law enforcement initiatives, and research included case studies, in-depth research on surviving victims, and methods to assess global prevalence [17]. This work was important in the identification, definition, and description of the phenomena. Reports suggested the wide range of sectors that employ trafficked labor, highlighted the suffering of victims, advanced law enforcement responses, and indicated the global magnitude of the problem. However, this work was of little benefit to prevention initiatives—which, from a public health perspective, are badly needed to make substantial population gains in the reduction of labor exploitation and its consequences.

However, investment in obtaining prevention evidence is growing. For instance, emerging findings from the field suggest that there may be limited benefit in “awareness-raising” interventions [16,18] and indicate possible unintended harm from training courses that are not solidly grounded in contextual evidence [19]. These findings confirm the need for a systematic integrated approach across the migration pathway that addresses structural conditions in addition to individual-level behaviors and risks [20–23].

To make genuine progress in prevention, we must begin by developing more robust evidence on what defines extreme forms of labor exploitation. For instance, various forms of exploitation (under the umbrella terms of “human trafficking” and “modern slavery”) have different population distributions, and each of these phenomena is likely to affect subgroups differently. Similarly, trafficking-related acts are very diverse, ranging from those related to forced sex work to abuses occurring in other sectors using forced and exploited labor, during which severe occupational hazards may occur [1,24].

Researchers urgently need to address intervention-focused questions about modifiable factors in the causal pathways to human trafficking in different contexts and for different populations [2]. Therefore, serious consideration must be given to the structures and practices that enable exploitation and leave individuals with extremely limited ability to alter their circumstances [16]. For example, complex structural factors exist and interact to drive labor exploitation, including growing income inequalities, the increasing power of corporations alongside diminishing power of workers, extortionate labor recruitment practices, and governance structures that favor businesses or employers over workers’ rights.

To begin the second generation of research and evaluation of what works to reduce exploitation, we need to move beyond focusing solely on individual behaviors to incorporate questions about how larger forces contribute to or prevent extreme exploitation. Emerging fields of intervention research include the examination of social protections, such as cash transfer schemes, transparent labor recruitment methods, worker-driven social responsibility reporting (as distinct from existing corporate social responsibility programs), and fairer labor immigration legislation in destination locations.

Trafficking research for prevention is still in the early stages. To achieve meaningful advancements, researchers and practitioners will have to work together to develop intervention frameworks that recognize the genuine complexity and real-world challenges of addressing human trafficking. Intervention and evaluation designs are needed that are grounded in evidence on the complexity of determinants and that specify their targeted populations and intended outcomes. Evaluations are required that monitor and document [25] the effects of interventions over time and across subpopulations and the ways in which these interventions operate toward their intended impact. Moreover, at this early stage in intervention research, investigators and implementers must leave space for regular learning and adaptation to course-correct programs and prevent unintended consequences. These types of dynamic evaluations can also respond to the appeal of realist evaluation, implementation science, or process evaluation to understand how, why, for whom, and under which circumstances interventions work in real-world settings [25–28].

We welcome the increase in well-intended calls for the use of experimental evaluation methods to address human trafficking. However, before interventions are subjected to resource-intensive evaluations, they will benefit from robust theories and implementation strategies that are grounded in evidence about causal processes and outcomes. Researchers should also consider if randomized trials are feasible, acceptable, and capable of answering questions of effectiveness for each specific intervention at its particular stage of development. Experimental designs may be extremely useful once developers, implementers, and evaluators have gathered sufficient evidence to be confident about the isolated contribution of an intervention to changes in the intended outcomes. Before then, resources need to be invested in the development of basic concepts, intervention theory, harm prevention, and appropriate research methods.

Future reductions in the global burden of labor exploitation will depend on researchers and practitioners working collaboratively to translate global good intentions into evidence-informed intervention designs. In this way, progress can extend beyond superficial patch-type responses to human trafficking and modern slavery in very diverse international settings and populations and address the deeper underlying drivers of this truly complex social problem.
